# Extremely varied phenotypes in granular corneal dystrophy type 2 heterozygotes

**Published:** 2012-06-27

**Authors:** Kyung Eun Han, Seung-il Choi, Woo Suk Chung, Se Hwan Jung, Nicholas Katsanis, Tae-im Kim, Eung Kweon Kim

**Affiliations:** 1 Corneal Dystrophy Research Institute, Yonsei University College of Medicine, Seoul, Korea; 2 Department of Ophthalmology, Yonsei University College of Medicine, Seoul, Korea; 3 Siloam Eye Hospital, Seoul, Korea; 4 Center for Human Disease Modeling, Duke University, Durham NC; 5 Severance Biomedical Science Institute, Yonsei University College of Medicine, Seoul, Korea; 6 Brain Korea 21 Project for Medical Science, Yonsei University College of Medicine, Seoul, Korea

## Abstract

**Purpose:**

To investigate the phenotypic variability of patients bearing the heterozygous R124H mutation in the *TGFBI* (transforming growth factor-beta-induced) gene that causes granular corneal dystrophy type 2 (GCD2).

**Methods:**

We describe the phenotypic range of GCD2 heterozygotes for the common R124H mutation in *TGFBI*; seven with an extremely mild phenotype and six with an extremely severe phenotype. Detailed slit-lamp photographs of these patients were generated. All patients had no history of ocular surgery and were diagnosed as being heterozygous for GCD2 by DNA analysis from peripheral blood. Expression levels of transforming growth factor-beta-induced protein (TGFBIp) were compared among cultured corneal fibroblasts from ten normal donors.

**Results:**

We report profound differences in the severity of the phenotype across our case series. Two patients with a mild phenotype were diagnosed as unaffected at presentation; however follow-up examinations revealed granular deposits. Importantly, we also observed familial clustering of phenotypic variance; five patients from two families with a mild phenotype showed a similarly mild phenotype within family members. Similarly, six patients from two families with severe phenotypes showed corneal deposits with similar patterns and severity within each distinct family, but distinct patterns between families. TGFBIp expressions from different donor derived cultured corneal fibroblasts were different between one another.

**Conclusions:**

GCD2 heterozygotes have extremely varied phenotypes between individual patients. However phenotypes were broadly consistent within families, suggesting that the observed variable expressivity might be regulated by other genetic factors that could influence the abundance of TGFBIp or the function of the pathway. From a clinical perspective, our data also highlighted that genetic analysis and meticulous slit-lamp examination in both eyes at multiple time intervals is necessary.

## Introduction

Granular corneal dystrophy type 2 (GCD2), also known as Avellino corneal dystrophy, is an autosomal dominant disorder caused by a mutation in codon 124 of the transforming growth factor-beta-induced (*TGFBI*) gene, in which histidine replaces arginine (R124H). Holland et al. [1] reported characteristics of deposits in GCD2 heterozygotes as being detectable early in life, from age 6 to 16, while granular deposits in the superficial stromal layer and lattice deposits in the deep stroma are detected later, over the age of 35. Diffuse anterior stromal haze can be observed in some patients over 50 years. Usually, granular and lattice corneal deposits slowly increase in size and number with age, and visual impairment occurs later in life. By contrast, the phenotype of GCD2 homozygotes has been reported as earlier onset, from age 3 to 5, with dense and confluent granular deposits which aggressively progress with age [2,3], indicative of a dosage effect.

Although the roles of the R124H mutation in generating GCD2 is now well documented, both prospective and retrospective clinical analyses have begun to uncover significant variability in clinical expressivity that is as yet poorly defined and understood. Konishi et al. [4] reported that two distinct opacity patterns were observed in GCD2 heterozygotes: the typical, common type as observed by Holland et al. [1], and a diffuse subepithelial opacity dominant type. Rosenwasser et al. [5] also reported that variable proportions of granular and lattice deposits, whether granular dominant or lattice dominant, were observed within the same family and among different families. However, the extent of variation has not been discussed widely, especially with regard to a) extreme cases; and b) comparisons of inter- versus intra-familial variability.

In this study, we draw from the phenotypic analysis of ~900 GCD2 heterozygotes and report the cases which represent the extreme ranges of the phenotype. In addition to documenting the phenotype with detailed slit-lamp photography, we also show significant familial clustering. This suggests that a shared biologic mechanism underpins variable expressivity. These observations will contribute to the improved diagnosis and management of patients, especially in the context of refractive surgery candidates. Further, our studies suggest that additional factors are likely to modulate the penetrance and expressivity of primary causal *TGFBI* mutations.

## Methods

### Clinical evaluation of patients

A total of 906 GCD2 heterozygotes, confirmed genetically with DNA sequencing from 160 unrelated families were examined and photographed with a slit-lamp camera (D2X, Nikon Corporation, Tokyo, Japan) from January 2006 to August 2011 at Yonsei University Medical Center, Seoul, Korea. DNA sequencing for the *TGFBI* locus was performed as reported [4, 6,7]. Genomic DNA was extracted from the peripheral leukocytes of the patients using a QIAamp DNA Blood Mini Kit (Qiagen, Hilden, Germany) according to the manufacturer’s protocol. Exon 4 for the R124H mutation of *TGFBI* was analyzed by polymerase chain reaction (PCR) amplification using Maxime PCR PreMix Kit (Cat. No. 25167; Gentaur, San Jose CA). The reaction mixture was amplified as follows: 5 min at 95 °C; 30 cycles of 94 °C for 30 s, 58 °C for 30 s, and 72 °C for 30 s; and 72 °C for 7 min. The oligonucleotide primers are listed in Table 1. Sequencing conditions were as follows; 90 s at 95 °C; 25 cycles of 95 °C for 30 s, 50 °C for 5 s, and 60 °C for 4 min. The products of the sequencing reaction were analyzed using a fluorescent ABI 3730 XL (Applied Biosystems, Foster City, CA). Nearly all the participants showed a typical phenotype of GCD2, with features of early onset granular deposits, late onset lattice deposits, and often diffuse haze in individuals older than 50. Within our cases, we found seven with an extremely mild phenotype and six with an extremely severe phenotype; since these represented the extreme phenotypes among our cases, they were selected for the present report. Clinical characteristics including age, gender, usage of eyeglasses or contact lenses, type of contact lens, occupation, and workplace were documented.

**Table 1:** PCR primer pairs.

**Accession number**	**Primer sequence (5'-3')**	**Product size (bp)**	**Annealing temp. (°C)**
NM_000358	Forward-CCCCAGAGGCCATCCCTCCT	224	58
	Reverse-CCGGGCAGACGGAGGTCATC		

### Immunoblot analysis

**Figure 1:**
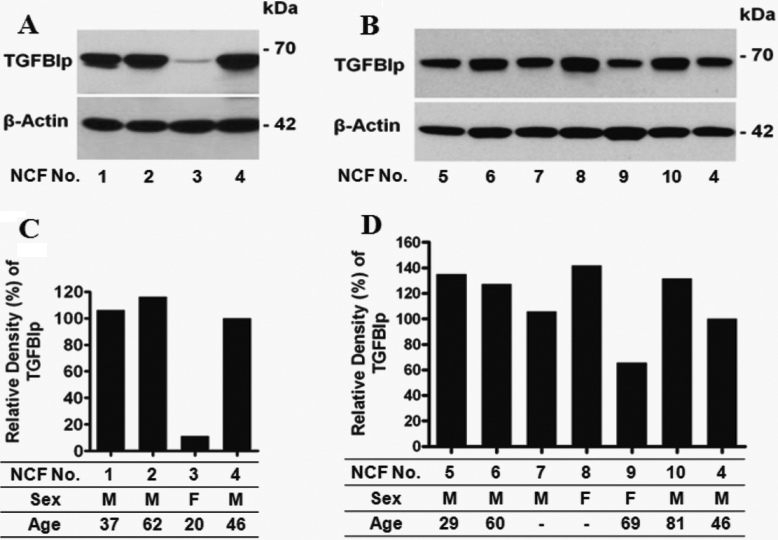
Expression of transforming growth factor-beta-induced-gene protein (TGFBIp) in ten different normal corneal fibroblasts (NCF). **A**, **B**: western blots show evidence of differential expression of TGFBIp in cultured NCF-1 (37 year-old man), NCF-2 (62 year-old man), NCF-3 (20 year-old woman), NCF-4 (46 year-old man), NCF-5 (29 year-old man), NCF-6 (60 year-old man), NCF-7 (man with unknown-age), NCF-8 (woman with unknown-age), NCF-9 (69 year-old woman), NCF-10 (81 year-old man) from normal donor corneas. In the second experiment, NCF-4 was reloaded for a comparison of the two separate experiments. **C**, **D**: Relative density of TGFBIp expression of **A** and **B**, respectively. Relative density of NCF-4 was adjusted to 100% in both **C** and **D** for a comparison of the densities of normal corneal fibroblasts in two separate experiments. Blots containing 50 μg total protein from normal corneal fibroblasts were incubated with anti-TGFBIp antibody. TGFBIp expression was assayed using anti-TGFBIp polyclonal goat antibodies. β-actin has been used as a control for equal loading. Numbers on the right correspond to the molecular weight markers in kilodaltons (kDa).

To find differences in the expression of transforming growth factor-beta-induced protein (TGFBIp), a major component of GCD2 corneal deposits, western blot analysis of primary cultured corneal fibroblasts was performed on ten different donated normal corneas. Corneas were collected from a 37 year-old man (normal corneal fibroblasts (NCF-1), a 62 year-old man (NCF-2), a 20 year-old woman (NCF-3), a 46 year-old man (NCF-4), a 29 year-old man (NCF-5), a 60 year-old man (NCF-6), a man with unknown-age (NCF-7), a woman with unknown-age (NCF-8), a 69 year-old woman (NCF-9), and an 81 year-old man (NCF-10). We first performed western blot analysis of four corneas (Figure 1A) to find out the variable expression level of TGFBIp from NCF-1, 2, 3, and 4 and the expression level was very low in NCF-3. We performed western blot analysis of another six corneas from normal donors (Figure 1B). Western blot analysis of NCF-4 was repeated in the second experiment to allow comparison between the two separate experiments.

To measure the level of TGFBIp expression, total cellular protein was electrophoresed on Tris-glycine SDS polyacrylamide gels. Equal amounts of proteins were then transferred onto polyvinylidene difluoride membranes (Millipore Corp., Bedford, MA), blocked in 5% dry milk in Tris-buffered saline containing Tween-20 (TBS-T) at room temperature for one hour, and then incubated with primary antibodies to TGFBIp (0.2 µg/ml; Cat. No. AF2935; R&D Systems, Minneapolis, MN) and β-actin (1:5,000 dilution; Cat. No. A-5441; Sigma, St Louis, MO) overnight at 4 °C. After three washes with TBS-T, blots were incubated with secondary antibodies conjugated to horseradish peroxidase at room temperature for 1 h. Horseradish peroxidase-linked anti-mouse IgG (1:5,000 dilution; Cat. No. 31460) or anti-goat IgG (1:5,000 dilution; Cat. No. 31430) were used as a secondary antibody (Pierce, Rockford, IL). Western blots were visualized using the enhanced chemiluminescence system (Pierce).

This study was approved by the Severance Hospital Institutional Review Board and informed consent was obtained from all subjects participating in this study.

## Results

### The extreme range of mild R124H phenotypes

#### Patient 1

**Figure 2:**
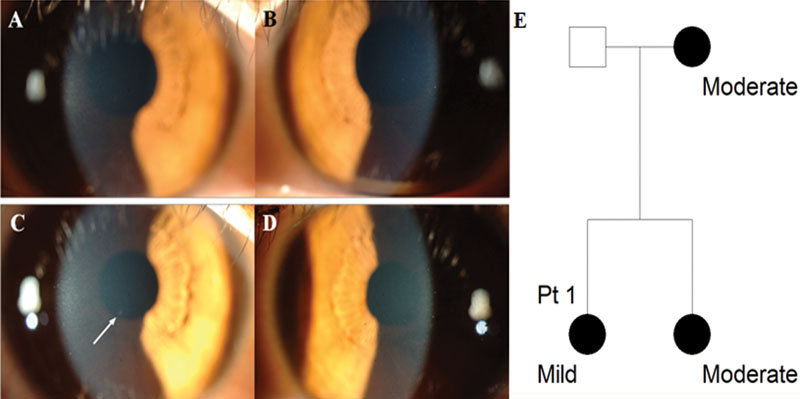
Slit-lamp photographs and pedigree of Patient 1 with mild phenotype, aged 23 years. **A**, **C**: Right eye, **B**, **D**: Left eye. **A**, **B**: At presentation, no corneal deposits were observed in both eyes. **C**, **D**: After six months, one faint corneal deposit was observed in the right eye (white arrow), but there was still no corneal deposit in the left eye. **E**: Pedigree of Patient 1. Circles represent women, squares represent men. The filled symbols indicate affected individuals.

A 48 year-old woman with several discrete granular corneal deposits visited our clinic with her daughter for evaluation of corneal dystrophy. Her 23 year-old daughter, determined to be a GCD2 R124H heterozygote, had best corrected visual acuity (BCVA) 20/20 in both eyes and showed no corneal deposit (Figure 2A,B). The patient had no history of glasses or contact lens wear. Upon reexamination six months later, the daughter showed one small corneal deposit in the right eye (white arrow, Figure 2C) and none in the left (Figure 2D).

#### Patient 2

A 28 year-old man was evaluated for corneal dystrophy because his older sister was diagnosed as being a GCD2 homozygote. He had used glasses for eight years. Upon initial examination, granular deposits or diffuse haziness were not observed in either eye (data not shown). Upon reexamination two years later, due to DNA analysis that showed him to be a GCD2 heterozygote, a newly developed, faint granular deposit was observed in each eye (data not shown).

#### Patients 3 and 4 (Mild family I)

**Figure 3:**
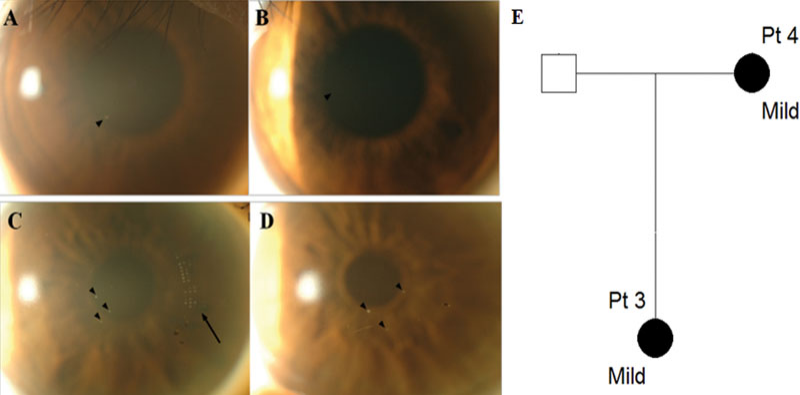
Slit-lamp photographs and pedigree of mild family I. **A**, **B**: Patient 3, aged 22; **C**, **D**: Patient 4, aged 47. **A**, **C**: Right eye, **B**, **D**: Left eye. **A**: Granular opacity was observed around pupil margin at 7 o’clock in the anterior stroma in the right eye (black arrowhead). **B**: Nearly non-detectable corneal deposit was observed in the left eye. **C**, **D**: A few dot-like granular deposits (black arrowheads) were observed centrally in the anterior stroma in both eyes. The black arrow in the right eye indicates the mark of a soft contact lens. **E**: Pedigree of mild family I. Circles represent women, squares represent men. The filled symbols indicate affected individuals.

A 22 year-old woman (Patient 3) presented with coincidentally found corneal deposits. She had used glasses for ten years and soft contact lenses for five years. On presentation, BCVA was 20/20 in the right eye and 20/25 in the left eye. A granular deposit was observed around the pupillary margin at 7 o’clock in the right eye and a faint opacity was observed at 9 o’clock in the left eye (black arrowheads, Figure 3A,B). Her mother, a 47 year-old woman (Patient 4), showed a few granular corneal deposits (Figure 3C,D). She had used soft contact lenses for approximately 14 h per day for 30 years.

#### Patients 5, 6, and 7 (Mild family II)

**Figure 4:**
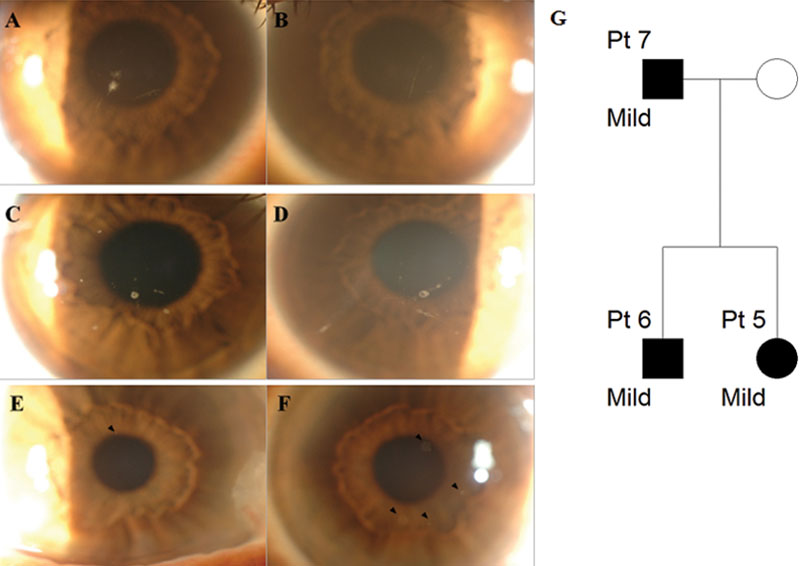
Slit-lamp photographs and pedigree of mild family II. **A**, **B**: Patient 5, aged 33; **C**, **D**: Patient 6, aged 34; **E**, **F**: Patient 7, aged 62. **A**, **C**, **E**: Right eye, **B**, **D**, **F**: Left eye. **A**: Several granular and linear corneal opacities were observed. **B**: A few small granular deposits and linear opacities were observed. **C**, **D**: Opacities of round and linear shapes were observed centrally in the anterior stroma in both eyes. **E**: A small granular deposit was observed around the pupillary margin at 12 o’clock (black arrowhead). **F**: Faint granular opacities at the 1, 4, 5, and 7 o’clock positions were observed (black arrowheads). **G**: Pedigree of mild family II. Circles represent women, squares represent men. The filled symbols indicate affected individuals.

A 33 year-old woman (Patient 5) was referred with minimal corneal deposits found preoperatively for refractive surgery. She had used glasses for 20 years, with rare contact lens use. Several granular deposits were observed in the center of the right eye and the left eye showed a few linear and granular deposits (Figure 4A,B). BCVA was 20/25 in the right eye and 20/30 in the left eye. Her 34 year-old brother (Patient 6) also had several corneal deposits (Figure 4C,D). He had used glasses for 20 years and had never used contact lenses. Her 62 year-old father (Patient 7) had one small faint granular deposit in the right eye and several in the left eye (black arrowheads, Figure 4E,F). He had no glasses or contact lens history.

### Patients with severe phenotypes

#### Patients 8, 9, and 10 (Severe family I)

**Figure 5:**
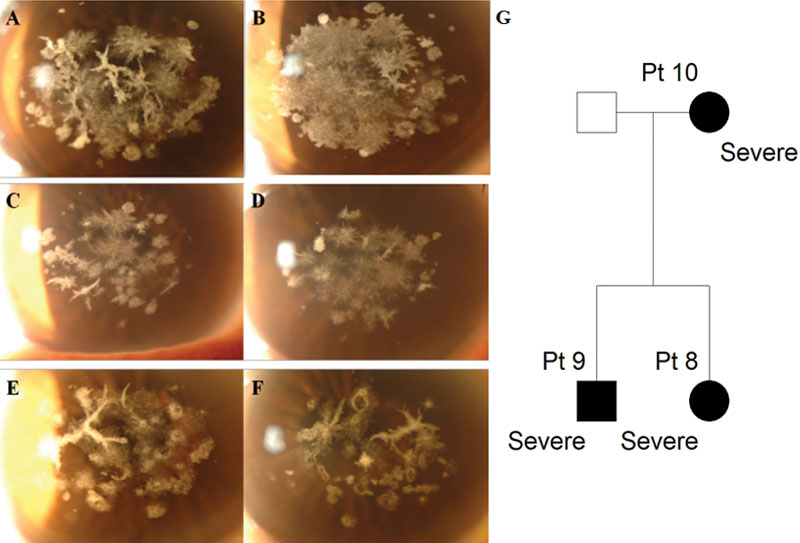
Slit-lamp photographs and pedigree of severe family I. **A**, **B**: Patient 8, aged 35; **C**, **D**: Patient 9, aged 38; **E**, **F**: Patient 10, aged 59. **A**, **C**, **E**: Right eye, **B**, **D**, **F**: Left eye. Confluent granular deposits, prominent lattice deposits, and anterior haze occupying the visual axis was observed in both eyes of all family members. They showed a more severe phenotype than other patients of a similar age and members within the family showed a similar pattern. **G**: Pedigree of severe family I. Circles represent women, squares represent men. The filled symbols indicate affected individuals.

A 35 year-old woman (Patient 8) was referred with multiple corneal deposits disturbing vision. Diffuse, confluent, crumb-shaped granular deposits occluded the visual axis in both eyes and numerous lattice deposits were also observed in the deep stroma (Figure 5A,B). BCVA was 20/40 in both eyes. She had no glasses or contact lens history. Her 38 year-old brother (Patient 9) had similar granular and lattice deposits (Figure 5C,D). BCVA was 20/40 in the right eye and 20/70 in the left eye; he had used glasses for 20 years. Their 59 year old mother (Patient 10) had similar, but less severe, corneal deposits than her daughter. She had used glasses for 30 years (Figure 5E,F).

#### Patients 11, 12, and 13 (Severe family II)

**Figure 6:**
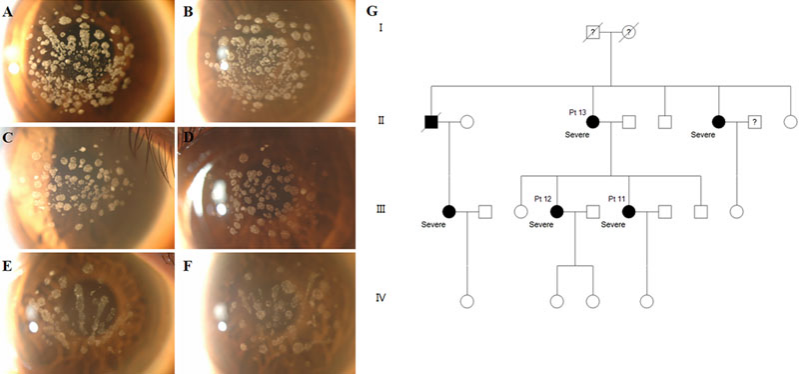
Slit-lamp photographs and pedigree of severe family II. **A**, **B**: Patient 11, aged 37; **C**, **D**: Patient 12, aged 40; **E**, **F**: Patient 13, aged 65. **A**, **C**, **E**: Right eye, **B**, **D**, **F**: Left eye. Numerous granular corneal deposits were observed in both eyes of all family members. They showed a more severe phenotype than other patients of a similar age and members within the family showed a similar pattern. **G**: Pedigree of severe family II. Circles represent women, squares represent men. The filled symbols indicate affected individuals.

A 37 year-old woman (Patient 11) was referred because of multiple corneal deposits and decreased visual acuity. Discrete, multiple, round, and ring-shaped granular deposits and a few lattice deposits were observed in both eyes (Figure 6A,B). Her sister and mother, age 40 (Patient 12) and 65 (Patient 13), respectively, had similar multiple granular deposits bilaterally (Figure 6C-F). Her sister had no glasses or contact lens history. BCVA was 20/25 in the right eye and 20/70 in the left eye. Her mother had used glasses for ten years. BCVA was 20/25 in the right eye and 20/40 in the left eye.

### Differential expression of TGFBIp among cultured corneal fibroblasts from different donors

The western blot showed TGFBIp expression as a specific protein band of expected size (~70 kDa) in all ten donor corneal fibroblasts. As shown in Figure 1, TGFBIp levels were differentially expressed among the normal corneal fibroblasts. The expression of TGFBIp was not related to patient age. The TGFBIp expression level detected was very low in NCF-3 (Figure 1A,C), while somewhat high in NCF-8 (Figure 1B,D). This result suggests that TGFBIp expression may be associated with the variety of GCD2 phenotypes.

## Discussion

Our findings illustrate the high degree of variability in the amount of deposits between GCD2 heterozygotes. No deposits could be found in a GCD2 positive 23 year-old woman (Patient 1) and 28 year-old man (Patient 2) and only a small number of corneal deposits were observed in other mild phenotype patients. By contrast, all six patients displaying the severe phenotype showed numerous deposits occupying nearly the entire central cornea, similar to homozygotes or patients having undergone refractive surgery [7,8].

Recently, the possibility of incomplete penetrance of GCD2 heterozygotes has been suggested. Cao et al. [9] reported reduced penetrance in seven patients, four of whom, aged 56, 37, 23, and 21, had no detectable corneal deposits, despite the presence of the R124H mutation. In that study, the existence of the deposits was evaluated with slit-lamp photographs presented to several third parties. Kim et al. [10] reported two cases of non-penetrance of GCD2 heterozygotes, aged 26 and 55 years, diagnosed by DNA analysis from epidermal keratinocytes obtained by adhesive tape. Our extremely mild phenotype patients (Patients 1 and 2), having no detectable deposits, could hardly be suspected of being heterozygotes before securing DNA diagnosis. Given the ages of these patients, and because their relatives showed definite GCD2 phenotypes, we failed to find even a small corneal deposit at initial examination even though we attempted to do so. However, follow-up of these patients showed faint, recognizable deposits on subsequent slit-lamp examinations.

In contrast, all the patients with a severe phenotype had excessive corneal deposits, which resulted in visual disturbance onset as early as the fourth decade (Patients 8, 9, and 11); they had no other history of ocular or systemic disease. Importantly, family members of patients displaying extremely severe phenotypes showed similarly severe patterns within families. However, the pattern of the disorder was family-specific. For example, the corneas of severe family I (Figure 5) had indistinct confluent granular deposits, prominent lattice deposits, and anterior haze in corneal stroma, while the affected corneas in severe family II (Figure 6) had numerous distinct granular deposits with a small number of lattice deposits.

The phenotypic range reported here presents a challenge for diagnosis and management due to the extreme variable penetrance and expressivity observed both by ourselves and other ophthalmologists. In our experience, the combination of genotypic data at the *TGFBI* locus and careful slit-lamp examinations at multiple time points are necessary to manage these patients accurately.

Our data also indicate that, within the observed phenotypic range, there is a distinct clustering of phenotypes within families which can be explained both by shared genetic factors and environmental exposure. In our study groups, we did not discern any extraordinary habits, including excessive outdoor activity (Table 2). Longitudinal studies of larger study groups will be required to investigate possible environmental contributions more thoroughly.

**Table 2:** Clinical characteristics of the patients.

**Patient No.**	**Severity**	**Gender**	**Age at presentation**	**Years of glasses wear**	**Years of contact lens wear**	**Lens type**	**BCVA OD**	**BCVA OS**	**Occupation**	**Working place**
1	Mild	F	23	-	-	-	20/20	20/20	Student	Indoor
2	Mild	M	28	8	-	-	NE	NE	Student	Indoor
3^a^	Mild	F	22	10	5	Soft	20/20	20/25	Student	Indoor
4	Mild	F	47	None	30	Soft	NE	NE	Office worker	Indoor
5^b^	Mild	F	33	20	almost not used	Soft	20/25	20/30	Student	Indoor
6	Mild	M	34	20	-	-	NE	NE	Programmer	Indoor
7	Mild	M	62	-	-	-	NE	NE	Carpenter	Outdoor
8^c^	Severe	F	35	-	-	-	20/40	20/40	Housewife	Indoor
9	Severe	M	38	20	-	-	20/40	20/70	Office worker	Indoor
10	Severe	F	59	30	-	-	20/70	20/40	NE	NE
11^d^	Severe	F	37	5	almost not used	Soft	20/25	20/70	Reporter	Outdoor/ Indoor
12	Severe	F	40	-	-	-	20/25	20/70	Private business	Indoor
13	Severe	F	65	10	-	-	20/25	20/40	Missionary	Indoor

^a^Patients 3 and 4 are family members; ^b^Patients 5, 6, and 7 are family members; ^c^Patients 8, 9, and 10 are family

members; ^d^Patients 11, 12, and 13 are family members; BCVA, best-corrected visual acuity; NE, not examined.

There is no established method to prevent the development or progression of corneal deposits in GCD2 patients so far. However, contact lenses have been suggested as a possible anti-aggravation factor. Severin et al. [11] and Rosters et al. [12] reported that soft contact lens wear had prevented the recurrence of granular deposits in eyes that had undergone penetrating keratoplasty for 14 years and 53 months, respectively. These authors hypothesized that the interaction between the contact lens and cornea altered corneal metabolism and resulted in a delay, or prevention, of recurrence [12]. In the present study, Patients 3 and 4 had been using soft contact lenses for 5 years and 30 years, respectively, and showed a mild phenotype which may be expected from the results of the previous study. However, other patients in this study with a mild phenotype had never used contact lenses (Table 2). Contact lens wear may be one of the factors in preventing the recurrence or progression of corneal deposits in certain patients, but the mild phenotype of virgin GCD2 heterozygotes in this study could not be explained solely by the use of contact lenses. Further studies would be helpful in determining the effect of contact lenses on corneal deposits.

In addition to the individual phenotype variations, we observed age-independent severity within families as some previous studies reported [1,5,13]. While aging is the most well known progression factor [1], the youngest family member (Patients 5, 8, and 11) in each family showed more severe expressivity in comparison to the oldest family member (Patients 7, 10, and 13), suggesting that the presence of an R124H mutation of the *TGFBI* gene and aging cannot solely explain phenotype severity.

In this study, we analyzed the expression of TGFBIp among ten different normal corneas and the expression of TGFBIp showed individual differences (Figure 1). The expression of TGFBIp was not related to patient age, because similar amounts of TGFBIp were expressed in both NCF-2 (aged 62) and NCF-4 (aged 46). These findings suggest expression differences of TGFBIp between GCD2 patients might exist and could serve as another possible reason for phenotype variation seen between both extremely mild and severe GCD2 patients and between individuals within families.

In conclusion, GCD2 heterozygotes have a highly variable phenotype, which can pose a challenge for securing clinical diagnosis, especially for mild cases. Our data suggest that offering prospective genetic screening in relatives of GCD2 patients has potential clinical value in securing sub-clinical phenotypes when coupled with meticulous slit-lamp examination and long-term observation.
